# Gastro-intestinal emergency surgery: Evaluation of morbidity and mortality. Protocol of a prospective, multicenter study in Italy for evaluating the burden of abdominal emergency surgery in different age groups. (The GESEMM study)

**DOI:** 10.3389/fsurg.2022.927044

**Published:** 2022-09-16

**Authors:** Gianluca Costa, Pietro Fransvea, Caterina Puccioni, Francesco Giovinazzo, Filippo Carannante, Gianfranco Bianco, Alberto Catamero, Gianluca Masciana, Valentina Miacci, Marco Caricato, Gabriella Teresa Capolupo, Gabriele Sganga

**Affiliations:** ^1^ Surgery Centre, Colorectal Surgery Unit, Fondazione Policlinico Universitariio Campus Bio-Medico, Università Campus Bio-Medico, Rome, Italy; ^2^ Emergency Surgery and Trauma, Fondazione Policlinico Universitario “A. Gemelli” IRCCS, Catholic University of Sacred Heart, Rome, Italy; ^3^ General Surgery and Liver Transplantation, Fondazione Policlinico Universitario A. Gemelli IRCCS, Rome, Italy

**Keywords:** acute care, surgery, gastrointestinal emergency, mortality, morbidity

## Abstract

Gastrointestinal emergencies (GE) are frequently encountered in emergency department (ED), and patients can present with wide-ranging symptoms. more than 3 million patients admitted to US hospitals each year for EGS diagnoses, more than the sum of all new cancer diagnoses. In addition to the complexity of the urgent surgical patient (often suffering from multiple co-morbidities), there is the unpredictability and the severity of the event. In the light of this, these patients need a rapid decision-making process that allows a correct diagnosis and an adequate and timely treatment. The primary endpoint of this Italian nationwide study is to analyze the clinicopathological findings, management strategies and short-term outcomes of gastrointestinal emergency procedures performed in patients over 18. Secondary endpoints will be to evaluate to analyze the prognostic role of existing risk-scores to define the most suitable scoring system for gastro-intestinal surgical emergency. The primary outcomes are 30-day overall postoperative morbidity and mortality rates. Secondary outcomes are 30-day postoperative morbidity and mortality rates, stratified for each procedure or cause of intervention, length of hospital stay, admission and length of stay in ICU, and place of discharge (home or rehabilitation or care facility). In conclusion, to improve the level of care that should be reserved for these patients, we aim to analyze the clinicopathological findings, management strategies and short-term outcomes of gastrointestinal emergency procedures performed in patients over 18, to analyze the prognostic role of existing risk-scores and to define new tools suitable for EGS. This process could ameliorate outcomes and avoid futile treatments. These results may potentially influence the survival of many high-risk EGS procedure.

## Introduction

Gastrointestinal emergencies (GE) are frequently encountered in emergency department (ED), and patients can present with wide-ranging symptoms (1–4). Symptoms that suggest an underlying GE can include: abdominal pain; nausea; vomiting; diarrhea; melaena; hematemesis; constipation; jaundice; and abdominal distension. The acute abdomen (AA) is a term given to sudden severe pain in the abdomen requiring fast diagnosis and treatment usually requiring emergency surgical procedures (5–7). Causes of AA may include: appendicitis; pancreatitis; peptic ulcer disease (PUD); gall bladder pathology; intestinal ischemia; diverticulitis; intestinal obstruction; and ruptured ectopic pregnancy. Emergency gastrointestinal surgery (EGS) is burdened by significant mortality and morbidity rates because it is performed with little to no advance planning or preparation, on patients who are in dire straits (8–10). Scott JW et al report that there are more than 3 million patients admitted to US hospitals each year for EGS diagnoses, more than the sum of all new cancer diagnoses (11). In addition to the complexity of the urgent surgical patient (often suffering from multiple co-morbidities), there is the unpredictability and the severity of the event. Frequently, it is necessary a rapid decision-making that allows a correct diagnosis and an adequate and timely treatment (12–14). Moreover, in other study Havens JM et al report that patient undergoing EGS operation are up to 8 times more likely to die postoperatively than are patients undergoing the same procedures electively (15). Furthermore, the increase in average life will lead more and more people over 65 to face surgical pathologies in an emergency setting. EGS in the elderly is characterized by a greater morbidity and mortality as well as by a global worsening of the residual quality of life (QoL) (16, 17). The explanation for the high percentage of acute complications could be found in the inevitable reduction of the functional reserve related to the age. An example is the reduction of the body's immune defenses in the humoral response of B cells, in the cell-mediated immune function and macrophage activity which explains the susceptibility to infectious complications, facilitated by the altered integrity of the skin barrier and mucous membranes too (18, 19). Any tool that can help the surgeon in the decision making process, could become very useful in order to reduce mortality and morbidity linked to the EGS (20–22). To do this, it is necessary to study the greatest number of risk factors associated with EGS, considering all age groups and all type of diseases.

## Protocol

### Objectives

The primary endpoint of this Italian nationwide study is to analyze the clinicopathological findings, management strategies and short-term outcomes of gastrointestinal emergency procedures performed in patients over 18. Secondary endpoints will be to evaluate to analyze the prognostic role of existing risk-scores to define the most suitable scoring system for gastro-intestinal surgical emergency. We will conduct an epidemiological investigation to gather information about the number of patients operated on yearly, and the prevalence of various pathological conditions leading patients to need emergency surgery. Furthermore, we aim to identify any specific parameters that may be used as variables for new scoring system, peri-operative variables predicting adverse results and any critical issues in the management of these patients.

### Study design and participating sites

The prospective, observational study will be conducted following a resident-led model, similar to what has been described by Banghu et al. and van Rossem et al. and the SPIRIT guidelines (23–25). Briefly, teams of medical students and surgical residents with senior staff surgeon oversight collect data on patients across Italy for 18 months. The Data Coordination Center (DCC) is the Colorectal Surgery Unit of Surgery Centre, – Fondazione Campus Bio-Medico University Hospital, University Campus Bio-Medico of Rome, Italy and the Study Director is responsible for the selection of the study sites. Any center performing emergency surgery can participate in this trial. The centers include academic medical centers, teaching hospitals, tertiary referral centers and community hospitals. Although this study protocol is similar to what adopted in previous research (26), in order to ensure that there is a uniformity of data acquisition, centers which do not participated in such previous study performed a 3-month pilot study collecting data retrospectively. All the data taken refers to the time of access of the patient in the emergency room or in any case to the first data available before any procedure. Such data are not published, and are stored separately for possible further analyses. All patients are treated according to the local hospital protocol and receive routine care as standard therapy. Whenever possible, patients with intestinal obstruction are treated according to SICUT Delphi consensus statements (27). The duration of the recruitment phase of patients is expected to be 18 months. The main strength of this project is the multicenter, prospective, contemporary methodology, with independent validation of data. This will produce high quality data on the emergency procedures carried out for gastro-intestinal emergency and on outcomes throughout Italy from a wide range of hospital types. Limitations include the inability to assess the postoperative visits to the general practitioner. Moreover, a minority of patients may present to other hospitals with complications following surgery, or because they need medical assessment. Despite this, teams will try to document the number of patients that were readmitted to other facilities. Our study uses the standard 30-day follow-up period, as this is the international standard and allows comparison to other studies. However, complications which may occur after 90 days will be reported as completely as possible.

## Trial population recruitment and eligibility criteria

### Inclusion criteria

All patients over the age of 18, undergoing urgent/emergency abdominal surgery will be included in the study. Emergency procedures are defined as unforeseen, non-elective operations according to the NCEPOD Classification of Interventions (28). The type of surgical approach takes into account open abdominal or laparoscopic procedures, including laparoscopic procedures that are converted to open abdominal procedures. Surgical procedures will be sorted on the basis of the 9th revision of International Classification of Disease Clinical Modification (ICD-9-CM). All abdominal procedures with ICD-9-CM code numbers ranging from 42.0 to 54.99 are considered eligible.

### Exclusion criteria

Exclusion criteria include any patient under 18 years at the day of surgery; lack of informed consent; patients already hospitalized and scheduled for the same procedure; participation in another trial.

### Outcome measures

The primary outcomes are 30-day overall postoperative morbidity and mortality rates. Secondary outcomes are 30-day postoperative morbidity and mortality rates, stratified for each procedure or cause of intervention, length of hospital stay, admission and length of stay in ICU, and place of discharge (home or rehabilitation or care facility). Severity of every disease will be assessed according to the AAST EGS score (29). Other secondary outcomes include the number of elderly subjects undergoing yearly emergency surgery, reported as the elderly to non-elderly patient ratio, emergency surgery in the elderly per 100,000 inhabitants, frequency of use of frailty score. Elderly are define as any patient >65 years old according to the World Health Organization. Moreover, the study will evaluate the sensitivity and specificity of the following scores: Charlson Age Comorbidity Index (CACI), Simplified Acute Physiology Score II (SAPSII), American College of Surgeons National Surgical Quality Improvement Program (ACS NSQIP), Calculation of Postoperative Risk in Emergency Surgery (CORES), Surgical Mortality Probability Model (SMPM), Urgent Surgery Elderly Mortality (USEM) score, Emergency Surgery Frailty index (EmSFI), 5 modified Frailty Index (5-mFI). The postoperative complications are reported and categorized according to the Clavien-Dindo classification system. Morbidity we will be assessed by detecting all adverse event according “Common terminology criteria for adverse events version 5” (30). Therefore, the Comprehensive Complication Index will be calculated (31).

### Data collection, validation, and management

In each participating hospital, one local investigator (usually a surgical resident/student) is responsible for data collection and for entering data into a password-protected electronic spreadsheet specifically constructed with predefined data fields. There are six categories, namely “patient demographics”, “comorbidities”, “clinicopathological data”, “surgical intervention”, “score”, and “follow-ups” (Table 1). Patient details will be recorded and anonymized using the code centre, an ID number and a unique alphanumeric code for any further integration. The anonymization procedure is provided by the enrolling centre. Patient data will be collected, if possible, on a daily basis; preoperative and intraoperative data will be processed after surgery, and the postoperative outcomes will be noted at the time of discharge and at the end of follow-ups. Data will be obtained from the electronic patient database, from admission charts, and operative reports, or directly from the surgeon who performed the operation when details were unclear or missing. Consent to participate in the study and to collect data for scientific purpose will be obtained from the patient at admission. The standardized data collection protocol has been approved by the Central Ethical Committee. There is no minimum number of patients per centre. Following data collection, only datasets with >95% data completeness will be accepted for pooled national analysis. The principal investigator (PI) at the selected site will identify an independent assessor to validate all data, with a target of >98% accuracy. Overall, at least 5% of the datasets will be independently validated. Outcome data will not be analyzed specific to each individual center. Data will be submitted monthly *via* e-mail or inserted in an online module. Once in the Data Coordination Centre (DCC) pooled warehouse, records are reviewed and edited and, whenever necessary, transformed to comply with the GESEMM data dictionary (see Table 1 for further information). The Study Director and the Study Coordinator will then identify unacceptable data entries using custom software queries to detect missing, impossible and improbable values and logical inconsistencies between data fields and across the forms. The DCC will then ask the sites to check for the incomplete data, and once the sites have resolved the data queries, the DCC will update the patient records. To identify complications during follow-up, each center will check their database to monitor visits to the emergency department, postoperative imaging or intervention, outpatient visits or hospital readmissions. Additional checking of admission diagnosis and surgical procedures in the study months will identify any missing patients. The data will be collected from each individual center according to the current Italian Law regarding privacy policy (Legislative Decree no. 196/2003 “RIGHT TO PERSONAL DATA PROTECTION CODE”). It will be the responsibility of the local investigators to ensure that the local data will be protected and held according to such privacy policy and in line with what has been approved by the ethics board. No patients are involved in setting the research question or the outcome measures; nor are they involved in the design and implementation of the study. There are no plans to involve patients in dissemination of results.

**Table 1:** Data spreadsheet fields: ASA American society of anaesthesiologists, BMI body mass index, BUN blood urea nitrogen, CPAP continuous positive airway pressure, CRP C-reactive protein, GCS glasgow coma scale, ICD-9-CM 9th revision of international classification of disease clinical modification, ID identifier, INR international normalized ratio, MI myocardial infarction, mFI modified frailty Index, PCI percutaneous coronary intervention, PLT platelet, WBC white blood cell, P-POSSUM portsmouth-physiological and operative severity score for the enUmeration of mortality and morbidity, CR-POSSUM coloRectal physiological and operative severity score for the enUmeration of mortality and morbidity, SAPS II simplified acute physiology score II, CACI charlson age-comorbidity index, EmSFI emergency surgical frailty index.

Form	Field	Options (definitions)
Demographics	ID	Progressive number
	ID center	Number
	ID code	Alphanumeric (3 characters)
	Age	In years
	Sex	Male/Female
	BMI	BMI in kg/m^2^
	Admission date	Day/month/year
	Operation date	Day/month/year
	Timing of surgery	Emergency/urgency
Clinicopathological data	Vital parameters	Systolic blood pressure, heart rate, respiratory rate, oxygen saturation, temperature, urine output, mechanical ventilation or CPAP, FiO2, GCS
	Laboratory analysis	Arterial blood gas analysis (PaO2, PaCO2 bicarbonate, lactates), chemistry (sodium, potassium, bilirubin, glycemia, CRP), renal function (BUN, creatinine), hemoglobin, WBC, PLT, INR
	Tumor	Site, TNM classification, Dukes staging system, grading, radicality of surgery, vascular invasion
Comorbidities	Associated diseases	Cardiovascular disease (ECG-report, hypertension, MI < 6 months, heart failure <30 days, chronic heart disease, Previous cardiac surgery or PCI, peripheral vasculopathy), cerebrovascular disease, respiratory disease (chronic lung diseases, respiratory failure), smoke, renal disease (acute/chronic), diabetes, liver disease (acute/chronic), solid tumor (localized/metastatic) leukemia, lymphoma, AIDS, drugs (oral anticoagulants, immunosuppressants or steroids, oral hypoglycemic agents or insulin), peptic ulcer
	Performance status	Hemiplegia, dementia, weight loss, physical activity, walk time, grip strength, exhaustion
Surgical intervention	Organ/body-district categories	Abdominal wall, appendicitis, biliary tract and pancreas, esophagus, large bowel, small bowel, solid organs, stomach and duodenum, thorax, Others
	Onset symptoms	Obstruction, acute abdomen (peritonitis—abscess and/or overt perforations), Vascular disorders, Trauma
	Primary operative indication	Benign/malignant/delayed elective
	Surgical approach	Open/Laparoscopic/Laparoscopic converted/Laparoscopic assisted
	Primary surgical procedure	ICD-9-CM code
	Associated procedures	Numbers
	List of associated procedures	ICD-9-CM code
	Intraoperative reliefs	Blood loss (ml), peritoneal contamination (yes/no)
	Operative time	Minutes
	ICU admission	Yes/no
	ICU length of stay	Dayes
Follow-ups	Date of discharge	Day/month/year
	Total length of stay	Days
	Type of discharge	Home, short-term rehabilitation facility, caregiver residential facility
	Complications 30-daypostoperatively	Yes/no
	Complication type	Free text
	Complication grade (Clavien-Dindo classification)	None/I/II/III/IV/V
	30-day mortality	Yes/no
Score		ASA, CACI, SAPSII, ACS-NSQIP, CORES, SMPM, USEM, EmSFI, 5-mFI

### Study time-line

The following timeline has been outlined, to define specific stages of the study:

• May 13–June 30, 2021: invitation to satellite centers to participate.
• 1 July–31 August, 2021: pilot study and standardization of data collection.
• 1 September 2021–31 August, 2022: main data collection.
• 1 September 2022–28 February, 2023: data collection on specific topics.
• 1 March–30 June, 2023: completion of the collection of any missing data.
• 1 July–31 December 2023: “interim analysis” of complete data excluding follow-up
• February 28, 2025: study completion for last potential follow-up.

### Statistical analysis

The report of this study will be prepared in accordance with guidelines set by the STROBE (Strengthening the Reporting of Observational Studies in Epidemiology) statement for observational studies (30). Statistical analysis will be performed either with SPSS software, version 21 to 26 (IBM Corp. Released 2020. IBM SPSS Statistics for MacOsx, Version 27.0. Armonk, NY: IBM Corp) for MacOSX or StataCorp2019 STATA Statistical Software: release 16 (College Station,TX: StataCorp LLC). First, data normality will be tested using the Shapiro–Wilk test or Kolmogorov– Smirnov test. Dichotomous data and counts will be presented in frequencies. Continuous data will be presented as mean values plus standard deviations, or as median values and interquartile ranges. The 95% confidence interval will always be reported where appropriate. Differences between means will be compared using the independent sample Student's *t* test, the pairwise comparison Student's *t* test, the Mann–Whitney *U* test, the Kruskal–Wallis test or other analysis of variance (ANOVA) tests. Differences between medians will be compared using the Kolmogorov–Smirnov test or special application of the Pearson chi square test by using the median as cut-off. To compare differences in frequencies, Fisher's exact test or *χ*^2^ test, with or without Yates correction will be performed. Receiver operating characteristic (ROC) curve analysis will be performed to estimate sensitivity and specificity of each score. Linear correlation will be assessed by Pearson's or Spearman's test, if needed. Multivariate analyses will be performed using logistic regression models that consider mortality and morbidity as dependent variables. A *P* value of <0.05 will be considered statistically significant.

### Expected results

We expect to obtain the following results:

• Identification of the mortality rate at 30 days (30-day mortality rate), divided according to the different type of intervention (ICD-9-CM classification) and stratified for 5-years age group;
• Identification of the general morbidity index at 30 days (30-day morbidity rate), divided according to the different type of intervention (ICD-9-CM classification) and stratified for 10-years age group;
• Identification of mean length of hospital stay (LOS), in days, according to the different surgical procedure group (ICD-9-CM classification);
• Sensitivity and specificity of the following scores:
  - Charlson Age Comorbidity Index (CACI)
  - Cohomprensive Complication Index
  - Simplified Acute Physiology Score II (SAPSII)
  - American College of Surgeons National Surgical Quality Improvement Program (ACS NSQIP)surgical risk calculator
  - Calculation of postOperative Risk in Emergency Surgery (CORES)
  - Surgical Mortality Probability Model (SMPM)
• Evaluation of the prevalence of emergency surgery in different age groups by geographic area (Emergency surgery per 100.000 inhabitants);
• Identification of an Elderly / Non Elderly ratio;
• Evaluation of the use of the following scores in elderly patients:
  - Frailty Fried Index
  - Canadian Study of Health and Aging (CSHA) frailty score
  - Emergency Surgery Frailty Index (EmSFI)
  - Urgent Surgery Elderly Mortality (USEM) score
  - 5 modified Frailty Index (5-mFI)

### Ethical aspects and ethics committee

The study will be conducted in accordance with the Declaration of Helsinki and respecting the guidelines on good clinical practice. At present, the study has been approved by the Ethical Committee of the coordinating center of Fondazione Policlinico Campus Bio-Medico of Rome [Prot.: PAR 87/21 (OSS)-ComEt UCBM] and by the Ethical committee of Fondazione Policlinico Universitario A. Gemelli of Rome ((ID:4639 No-Profit Study). The study protocol has been register on Clinicaltrials.gov. (ClinicalTrials.gov Identifier: NCT05226221).

### Privacy

Data will be collected by each individual center anonymously according to the current privacy regulations (Annex/Art. 13 of Legislative Decree 196/2003 “CODE REGARDING THE PROTECTION OF PERSONAL DATA”), making use of a progressive identification number and a unique code for any patient. All data collection forms will be sent to a non-medical collaborator who will accumulate them in a single general database, ensuring the anonymity of the patients and the center that sent them. The data and their processing phase will be owned by the center promoting the Study, but at the disposal of the Principal Investigator of each center.

### Publication policy and communication of results

The results of the GESEMM study will be disseminated through national and international conference presentations and peer-reviewed journals. The results will also be available through the study record website at ClinicalTrials.gov. Furthermore, additional studies and publications could be performed that analyse specific aspects of the data that will be presented. We are committed to ensuring that appropriate recognition is given to everyone who works on the study.

## Discussion

Emergency gastrointestinal surgery (EGS) is challenging in terms of decision-making, managing co-morbidity and post-operative rehabilitation with substatntial morbidity and mortality rate (31). On the light of this, it is pivotal to define the possible clinical-pathological features, pathways and treatment for gastrointestinal emergency (GE). Moreover, approximately half of all patients undergoing EGS will develop a postoperative complication, and up to 15% will be readmitted to the hospital within 30 days of their surgery (32). More recent pathophysiology knowledges and together with improved surgical and anaesthesiologic techniques allowed the surgeon to achieve better results in treating these high-risk patients (33, 34). However, diagnosis and treatment of GE still remain a challenge. In patients with GE, early warning scores (EWS) associated with abdominal signs and symptoms such as abdominal pain and tenderness can screen for patients needing prompt surgical procedures (35). EWS employ physiological, easy-to measure parameters, assessing variables such as systolic blood pressure, pulse rate, respiratory rate, temperature, oxygen saturations, and level of consciousness (36–39). However, physiological parameters are often not sufficient for risk stratification, as the GE involves a wide range of patients of different ages, with different disease, different morbidities and therefore different functional reserves. In the light of this, conceding the clinical importance of GE, considerable research is directed at identifying biomarkers suitable in predicting the severity and outcomes. Starting from previous researches, we have undertaken this project particularly focusing on reporting which are the EGS procedures that account for the greatest number of cases, deaths, complications, and inpatient cost; on the age-related and disease-related clinical differences as independent risk factor for the main clinical course; on the sensitivity and specificity of actual score in predicting EGS outcomes.

## Conclusion

Presently in Italy, recommendations to guide evaluation patients requiring EGS are not-homogeneous. In this article we present a protocol for a nationwide study designed to investigate the population undergoing EGS. To improve the level of care that should be reserved for these patients, we aim to analyse the clinicopathological findings, management strategies and short-term outcomes of gastrointestinal emergency procedures performed in patients over 18, to analyze the prognostic role of existing risk-scores and to define new tools suitable for EGS. This process could ameliorate outcomes and avoid futile treatments. These results may potentially influence the survival of many high-risk EGS procedure.

## Author contributions

GC, PF: Study conception and design, literature search, acquisition, interpretation and analysis of data, drafting and critically revising the article for important intellectual content, and final approval of the version to be published. FC, CP: literature search, acquisition, interpretation and analysis of data SM: literature search, acquisition, interpretation and analysis of data. LL,GTC,FC: acquisition, interpretation and analysis of data, drafting and critically revising the article for important intellectual content and final approval of the version to be published FG: acquisition, interpretation and analysis of data PM: acquisition, interpretation and analysis of data. GC,GS: drafting and critically revising the article for important intellectual content and final approval of the version to be published. All authors contributed to the article and approved the submitted version.

## Appendix: list of Italian Group for Gastro-Intestinal Surgery Postoperative Surveillance (IGo-GIPS)

Agresta F, Alemanno G, Altieri G, Antropoli M, Argenio G, Atzeni J, Avenia N, Azzinnaro A, Badessi G, Baldazzi G, Bergamini C, Biloslavo A, Bombardini C, Borzellino G, Bozzo S, Brachini G, Brisinda G, Buonanno GM, Canini T, Capolupo GT, Carannante F, Cardella S, Caricato M, Carrara G, Cascone Ca, Cassini D, Castriconi M, Catarci M, Ceccarelli G, Celi D, Ceresoli M, Chiarugi M, Cimino F, Cirocchi R, Cobuccio L, Coccolini C, Cocorullo G, Colangelo E, Colozzi S, Cortese F, Costa A, Costa G, Cozza V, Crucitti A, Cucinotta E, D'Alessio R, Dalla Caneva *P*, De Manzini N, de Manzoni Garberini A, De Nisco C, De Prizio M, De Sol A, De Stefano M, Dibella A, Di Cosimi C, Di Grezia M, Falcioni T, Falco N, Farina C, Fico V, Finotti E, Fontana T, Francioni G, Fransvea *P*, Frezza B, Garbarino GM, Garulli G, Genna M, Giannessi S, Gioffrè A, Giordano A, Gozzo D, Grimaldi S, Iacopini V, Iarussi T, Kurihara H, La Greca A, Laracca GG, Laterza E, La Vaccara V, Leonardi A, Lepre L, Liotta G, Luridiana G, Magalini S, Malagnino A, Mar G, Mariani D, Marini *P*, Marzaioli R, Macianà G, Mazzoni G, Mecarelli V, Mercantini *P*, Mingoli A, Mirco *P*, Montuori M, Nigro C, Occhionorelli S, Paderno N, Palini GM, Paradies D, Paroli M, Perrone F, Pepe G, Petruzzelli L, Pezzolla A, Piazza D, Piazza V, Pignata G, Pinotti E, Pisanu A, Podda M, Poillucci G, Porfidia R, Puccioni C, Rocca A, Rondelli F, Rossi G, Sacchi M, Sapienza *P*, Sganga G, Spagnoli A, Spinoglio G, Sulis R, Tartaglia D, Tranà C, Travaglino A, Tomaiuolo *P*, Tomassini F, Tropeano G, Valeri A, Zago M, Zanoni E.
